# Review of the Current State of Artificial Intelligence in Pediatric Cardiovascular Magnetic Resonance Imaging

**DOI:** 10.3390/children12040416

**Published:** 2025-03-26

**Authors:** Addison Gearhart, Scott Anjewierden, Sujatha Buddhe, Animesh Tandon

**Affiliations:** 1 Department of Cardiology, Seattle Children’s Hospital, Seattle, WA 98105, USA; 2 Department of Pediatrics, University of Washington, Seattle, WA 98195, USA; 3 Division of Pediatric Cardiology, University of Utah, Salt Lake City, UT 84112, USA; 4 Division of Pediatric Cardiology, School of Medicine, Stanford University, Stanford, CA 94305, USA; 5 Department of Heart, Vascular and Thoracic, Division of Cardiology and Cardiovascular Medicine, Children’s Institute, Cleveland Clinic, Cleveland, OH 44195, USA; 6 Cleveland Clinic Children’s Center for Artificial Intelligence (C4AI), Cleveland Clinic Children’s, Cleveland, OH 44195, USA; 7 Department of Pediatrics, Cleveland Clinic Lerner College of Medicine of Case Western Reserve University, Cleveland, OH 44106, USA

**Keywords:** congenital heart disease, artificial intelligence, cardiac magnetic resonance imaging

## Abstract

Cardiovascular magnetic resonance (CMR) imaging is essential for the management of congenital heart disease (CHD), due to the ability to perform anatomic and physiologic assessments of patients. However, CMR scans can be time-consuming to perform and analyze, creating roadblocks to broader use of CMR in CHD. Recent publications have shown artificial intelligence (AI) has the potential to increase efficiency, improve image quality, and reduce errors. This review examines the use of AI techniques to improve CMR in CHD, by focusing on deep learning techniques applied to image acquisition and reconstruction, image processing and reporting, clinical use cases, and future directions.

## 1. Introduction

**Figure 1:**
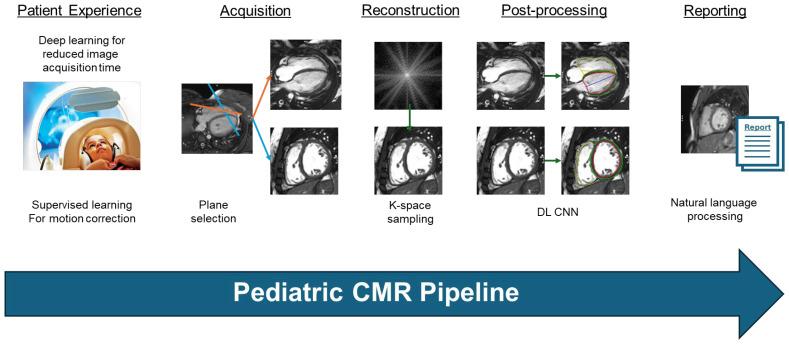
Proposed pipeline for AI in pediatric CMR pipeline.

Cardiovascular magnetic resonance (CMR) imaging for pediatric and congenital cardiology has been a rapidly growing field due to technical advancements over the past few decades, which have enabled it to be the most thorough non-invasive imaging technique for assessing structural heart disease, cardiovascular hemodynamics, tissue characterization, and perfusion. Data gleaned from examinations guide clinical diagnosis, medical and surgical planning, and risk and outcome prediction. Pediatric CMR, however, is resource-intensive, often limiting its widespread use. Barriers include limited availability due to the need to share the CMR scanner with other departments, the need for sedation in younger or special needs children, high costs, length of examinations, time spent on post-processing, and expertise in performing, interpreting, and reading complex congenital heart disease (CHD) scans. As such, it is not surprising that there has been a continued and growing interest in applying artificial intelligence (AI) to the acquisition and interpretation of CMR to improve the quality and efficiency of these examinations. Furthermore, from a research perspective, the pediatric cardiology community has become increasingly excited by the prospect for AI to vastly improve research in pediatric CMR through the augmentation and storage of datasets in diseases historically hindered by rarity and small patient numbers. Recent publications, particularly in adult literature, have shown the success of AI in overcoming many of the cited limitations that similarly hinder progress in pediatric cardiology. The continued challenge for advancing AI in pediatric CMR remains the translation from research work to clinical incorporation and use. Few prior reviews have focused on the use of AI in pediatric CMR to improve specific aspects of the pediatric CMR pipeline [1], with the majority of reviews centered on adult CMR [2,3]. This review article summarizes pediatric-specific AI in CMR acquisition and reconstruction, AI in image processing and reporting, AI in clinical use today, and lastly introduces potential future applications of AI in pediatric CMR (Figure 1).

## 2. AI in CMR Acquisition and Reconstruction

### 2.1. Acquisition

Efficient image acquisition is essential in pediatric cardiac MRI, where maintaining patient cooperation and minimizing sedation is critical. AI has significantly advanced this process by automating and optimizing imaging protocols. The potential benefits, challenges, and proposed mechanisms of AI in pediatric CMR are reviewed in Table 1. Various data acquisition and image reconstruction techniques have been developed to generate high-quality images from undersampled data, enhancing the speed and accuracy of pediatric cardiac MRI.

AI algorithms can recommend or automatically select imaging sequences. For instance, Alansary et al. demonstrated that multi-scale search strategies outperform fixed-scale agents in images with large fields of view and noisy backgrounds, such as cardiac MRI [3]. They evaluated several deep Q-network (DQN) architectures for detecting multiple landmarks using three different medical imaging datasets: fetal head ultrasound (US), adult brain, and cardiac magnetic resonance imaging (MRI). This approach significantly enhances performance and accelerates the search process by 4–5 times using hierarchical action steps that alternate between larger and smaller steps for fine-tuning.

Pediatric patients often face challenges in remaining still during MRI scans, leading to motion artifacts that degrade image quality. AI techniques, such as supervised learning models, can detect and correct motion artifacts in real time. Lyu et al. showed that their method reliably generates missing intermediate frames based on adjacent frames, improving the temporal resolution of cine cardiac MRI sequences [4]. Their model utilizes bi-directional convolutional long short-term memory (ConvLSTM) and multi-scale convolutions to improve the performance of the proposed network, in which bi-directional ConvLSTMs handle long-range temporal features while multi-scale convolutions gather both local and global features. Similarly, Küstner et al. developed a deep-learning-based super-resolution framework for motion-compensated isotropic 3D coronary MR angiography (CMRA), enabling free-breathing acquisitions in under a minute [5]. A 16-fold increase in spatial resolution was achieved by reconstructing a high-resolution (HR) isotropic CMRA (0.9 mm^3^ or 1.2 mm^3^) from a low-resolution (LR) anisotropic CMRA (0.9 × 3.6 × 3.6 mm^3^ or 1.2 × 4.8 × 4.8 mm^3^).

Four-dimensional (4D) flow is an increasingly used sequence to characterize flow through multiple valves and vessels in patients with CHD [6]. Because of the increased time to acquire flow values across the whole chest, rather than 2–4 two-dimensional flow images, many groups are working on improving the acquisition time of 4D flow datasets through super-resolution [7].

### 2.2. Two-Dimensional Reconstructions

The computationally intensive process of image reconstruction, which converts raw MRI data into clinically interpretable images, has also benefited from AI advancements. Deep learning (DL) sequences have accelerated cardiac MRI (CMR) imaging by enabling faster acquisition while preserving image quality. For example, Klemenz et al. showed that DL-based cine sequences achieve optimal compromise with no loss of image quality or volumetric accuracy, reducing acquisition time by over 50% [8]. In their study, total acquisition time was significantly lower (median) for a short-axis stack for 1RR cine, 3 RR cine, and 6RR cine, compared to the standard sequence. Volumetric results showed no difference for the conventional cine and 3RR cine, but the 1RR cine sequence significantly underestimated EF because of a different segmentation of the papillary muscles. Pednekar et al. evaluated compressed sensitivity encoding (C-SENSE) with systematic undersampling, demonstrating comparable biventricular volumetric indices up to an undersampling factor of acceleration/undersampling factor (R) = 5 [9]. They found that there was a significant decrease in the quality imperceptibility (QI) measures and edge definition scores as R increased.

AI-based denoising and reconstruction techniques have also significantly improved image quality. Vollbrecht et al. reported that deep learning (DL)-based denoising outperformed conventional compressed sensing (CS) for fetal cardiac cine imaging in congenital heart disease (CHD), producing superior image quality [10]. In their study, images were reconstructed using both compressed sensing (bSSFP CS) and a pre-trained convolutional neural network trained for DL denoising (bSSFP DL). In another study employed by Zucker et al., a DL algorithm was used to reconstruct 12-fold accelerated balanced steady-state free precession (bSSFP) cardiac cine MRI images, achieving substantially shorter acquisition times with slightly reduced image quality [11].

### 2.3. Three-Dimensional Reconstructions

Reconstruction of cardiac anatomy into three-dimensional (3D) models that can be used for surgical planning or converted into virtual reality or 3D-printed anatomical models is a key part of CMR post-processing. Although much of the literature focuses on the segmentation of cardiac CT datasets into 3D models, similar tools can be used for magnetic resonance angiograms or 3D whole heart sequences [12]. Commercial tools today contain manual and semi-automated methods, but advancements are being made in automated methods [13,14,15,16]. Recently, an open-source dataset was released to improve the development of these automated tools [17].

## 3. AI in Image Processing and Reporting

### 3.1. Volumetric Analysis

AI has further contributed to automated and standardized image analysis. Post-processing CMR datasets is a time-consuming but necessary step in CMR. Most clinical tools in use today have some element of automated segmentation tools built in, initially using rules-based algorithms, and more recently moving to DL. These tools have improved the workflow for performing volumetric calculations in CMR, and the quality of the automated segmentations is generally acceptable to clinicians [18]. However, these tools are not generally optimized for patients with CHD [19], leaving post-processing a significant burden [20]. In particular, though this has not been studied extensively, logically the more the ventricular shape and morphology diverge from “normal”, the more inaccurate algorithms trained on “normal” ventricles are likely to be. For instance, there was significant inaccuracy in a commercially available convolutional neural network (CNN)-based algorithm used to segment cases of repaired tetralogy of Fallot, with errors in slice selection, phase selection, and edge detection [21,22]. More recently, disease-specific segmentations are showing promise, though these would possibly have limited applicability for general clinical use [19,21,22]. A more recent study, examining a three-dimensional CNN built specifically for repaired tetralogy of Fallot performed well, but best when trained on both control and tetralogy datasets [22]. There are other examples of a CNN (ResNet50) used for contouring for rTOF [23]. Yao et al. developed an end-to-end DL pipeline for automated ventricular segmentation in cardiac MRI data from patients with Fontan circulation. This approach provides fast, standardized segmentation across multiple centers [13]. However, disease-specific segmentation algorithms for the vast majority of CHD lesions have yet to be built or limit the full translation for general clinical use.

### 3.2. 2 Dimensional Flow Quantification

Generally, flow quantification in 2D-phase contrast imaging is less time-consuming and complicated, because much of the work to acquire the optimal image is performed at the time of the scan being performed, namely planning a flow plane orthogonal to the vessel of interest. Most current clinical post-processing tools use automated methods, either with edge detection or with DL (e.g., U-net) [24]. Oscanoa et al. showed that complex-difference deep learning (CD-DL) reconstruction produced quantitative 2D phase-contrast MRI measurements with ≤5% error in accuracy and precision at up to 9× acceleration, demonstrating clinical feasibility in pediatric cohorts [25].

### 3.3. 4 Dimensional Flow Quantification

Four-dimensional (4D) flow, is a sequence that is being used with increasing frequency and is acquired by placing a volume over the anatomy of interest and obtaining flow in three dimensions, over time [26], and can provide significant information about specific disease states [27]. Obtaining flow over a three-dimensional volume, however, pushes the burden of flow quantification into post-processing, which can also be time-consuming. Now, deep-learning and AI approaches [7] are being developed to improve processing for these datasets, including 3D U-Nets for general segmentation [28] and via commercial tools for valve tracking [29]. In addition, some DL approaches are being developed to calculate volumetrics and flow simultaneously from 4D flow datasets using a variety of 2D and 3D U-Nets [30].

### 3.4. CMR Reporting

To our knowledge, there are no reports to date of the use of AI tools for CMR reporting in CHD. However, in the current era, the development of large language models and other foundation models not only enables text-to-text generation but may allow direct image-to-text generation of congenital CMR reports. Furthermore, the Society for Cardiovascular Magnetic Resonance Pediatric and Congenital Heart Disease has recently formed a working group focused on structured reporting, which may allow increased use of AI in CHD CMR through improved image annotation and allow easier multicenter analysis.

## 4. AI in Clinical Use Today

### Scanner Integration in the Clinical Setting

More recently, AI software has become integrated into many commercially available MRI scanners. In the United States, these AI algorithms must be approved by the Food and Drug Administration (FDA), which considers software as a medical device. These algorithms go through the same approval pathways as traditional medical devices within the United States, though recently there has been some concern that there are no uniform guidelines set by the FDA to regulate these devices [31]. There are currently over 1000 AI/ML-enabled medical devices approved by the FDA, with a small number of these algorithms designed for use with CMR [32,33]. While the specific details of these algorithms are typically considered important intellectual property for the companies that own them, some information on the clinical utility of the algorithms is often available.

Many of the newest commercially available MRI scanners now boast FDA-approved AI algorithms integrated directly into their platform. For example, Philips (Philips Healthcare, Best, Netherlands) now markets “Philips SmartSpeed Cardiac”, which is an AI algorithm applied at the beginning of the MR reconstruction chain to denoise and achieve significantly faster scanning times compared to conventional MR scans [33,34]. Siemens (Siemens Healthineers, Forchheim, Germany) has recently partnered with HeartVista (HeartVista Inc., Los Altos, CA, USA), a company that developed AI-guided acquisition software with similar claims of shortened exam times and improved image consistency [35]. Additionally, some companies have been focusing on offering cloud-based software that provides an AI-based solution for CMR image analysis. This offers the benefit of simplified integration into existing clinical workflows by uploading the DICOM study. AI4MedImaging is a company with an FDA-approved and CE-marked algorithm, “AI4CMR”, that can perform a fully automatic cardiac segmentation for volume quantification [36,37].

Despite this growing field of AI algorithms with regulatory approval for clinical implementation, there are not any algorithms that are specifically trained for or tested on children with CHD. Vollbrecht et al. [10], recently evaluated the commercially available DL algorithm (SmartSpeed AI, Philips Healthcare). This algorithm merges a compressed sensing approach with a convolutional neural network-based sparsification approach to improve resolution and reduce noise. This particular algorithm is not specific to the anatomy investigated and applies across numerous MR applications including CHD and in the case of this study fetal cardiac MRI. In this study, DL-based denoising reconstruction provided a higher apparent signal-to-noise ratio, a higher contrast-to-noise ratio, and improved diagnostic confidence when assessing cardiovascular structures compared to standard reconstruction.

This study provides an example of where advancements in AI-enabled CMR for adults (such as DL-based denoising) can benefit the subpopulation of children and those with CHD. This is particularly important as the heterogeneity and complexity of the anatomy in these patients often leads to a very high burden for physicians responsible for processing and interpreting these studies [20]. However, in cases such as automated image segmentation, the technology does not have the same degree of generalizability as previously discussed in this article [19,20,37,38].

Multi-institutional collaboration and partnerships between academia and industry will be required to translate AI algorithms into clinical practice for children and individuals with CHD. Within the last year, the first publicly available CMR dataset for whole-heart segmentation in patients with CHD was made available [17], and similar collaborative efforts will be important moving forward. For example, the FORCE Fontan registry, a cloud-based platform for data aggregation and AI analytics, now includes 42 centers, 4200 patients, 6400 CMRs, and patient outcome data enabling collaborative and accelerated AI research for this vulnerable population [38]. A number of other AI in pediatric cardiology working groups are fostering collaboration to accrue CHD datasets to facilitate more research.

## 5. Future Directions

### 5.1. Increasing Computational Power and Pediatric Specific Datasets

Limitations that slow the advancement of efforts to incorporate AI into the pediatric CMR clinical setting include the need for more computational power. Reconstruction and storage of datasets come at a financial cost. Current efforts to mitigate these challenges include coil compression, stochastic gradient descent, and randomized sketching algorithms. Specific to pediatrics, another limitation remains that many algorithms being developed are adult-focused. This is due in part to demand as well as notably reduced access to fully sampled ground-truth pediatric datasets for training. Efforts to overcome this include techniques such as the use of a Deep Convolutional Generative Adversarial Network (DCGAN) that produces synthetically segmented CMR images and strengthens the training data [39]. Recent advancements in this technique have shown promise in the ability of deep-learning algorithms to successfully and accurately segment CMR images of complex CHD subjects beyond the existing AI methods using both generative adversarial network (GAN) to synthetically augment the training dataset via generating synthetic CMR images and their corresponding chamber segmentations, as well as fully convolutional neural networks trained on only 64 patients with CHD [40].

### 5.2. Accelerated Image Acquisition and Reconstruction

Clinically, there is a strong need for shortened scan times and efficient scan protocols in pediatric CMR. Pediatric patients have a limited ability to cooperate for the entirety of the exam particularly to comply with the breath holds and need to stay still. As such, many require anesthesia. Utilizing AI to improve the time it takes to complete a pediatric CMR could increase the efficiency and comfort of the examinations, reduce the length of exposure and risks of anesthesia, increase the availability of scan time, and ultimately reduce costs. CMR examinations are naturally slow due to the need to acquire multiple lines of k-space, and the need for ECG synchronization and breath holds. Recent publications utilizing DL to overcome the length of examinations often without compromising quality include the acquisition of images at low resolution with or without under-sampling and then retrospectively reconstructing the images at higher resolution using DL-based super-resolution techniques such as a sonic DL method with an acceleration factor [41]. New approaches include a volumetric approach of using a 4D flow scan with DL image reconstruction that shows promise in pediatrics because it comprehensively provides flow, functional, and anatomical data with a single sequence acquisition obviating the potential need for performing numerous conventional length scans [26]. The DL automatically detects the landmarks of the heart and then performs the reformatting of these images to provide standard views obtained on conventional scans giving the interpreting clinician the ability to view the whole chest from any desired plane [17]. AI has also been used in real-time to conventional 2D imaging using free breathing with respiratory triggering in pediatrics to accelerate the examination and make the exam less difficult on the patient, potentially removing the need for anesthesia. Other groups have used AI to reconstruct free breathing and ungated CMR data from undersampled measurements [9,42,43].

### 5.3. Segmentation

Although existing AI algorithms in commonly available post-processing software for ventricular volume and functional measurements and flow calculations perform better for structurally normal hearts, newer AI algorithms show promise. Karimi-Bidhendi et al. showed that a fully convolutional network could be applied successfully in pediatric patients with CHD with superior performance to the algorithms clinically used in a commercially available platform [40]. Furthermore, a DL convolutional neural network pipeline for automated contouring and assessment of ventricular volumes in single ventricle Fontan patients across multiple centers has been published and shown to be accurate [13].

### 5.4. Radiomics for Risk Prediction and Disease Detection

An emerging area of interest between the interface of AI and CMR is radiomics. Radiomics extracts thousands of quantitative features undetectable to the human eye from medical images, which can subsequently be analyzed by DL techniques to generate knowledge, define novel biomarkers, assist with outcome prediction or aid in decision-making. Proponents state it may offer promise for delivering a more personalized healthcare delivery model. Early success using standard logistic regression of radiomics derived from late gadolinium enhancement in hypertrophic cardiomyopathy has shown the benefit of sudden cardiac risk detection beyond standard risk models [44]. Furthermore, another AI model built from the UK Biobank adding CMR radiomic indices to traditional vascular risk factors, and conventional CMR metrics aided in the prediction of atrial fibrillation, heart failure, myocardial infarction, and stroke [45]. A radiomics-based CMR model has shown promise with one vs. rest ROC-AUC of 0.95 for the automated diagnosis of left ventricular noncompaction, hypertrophic cardiomyopathy, and dilated cardiomyopathy [46]. While its use at present has been primarily dedicated to adult disease, some pediatric groups have started to apply this research to CHD.

### 5.5. Disease Diagnosis

The future ideal AI-integrated CMR system for clinical deployment should be seamlessly incorporated from acquisition at the scanner to the ultimate diagnosis of the CHD or acquired heart disease. The particular challenge for pediatrics continues to be the vast heterogeneity of CHD, range of age spans, and racial/ethnic groups that need to be adequately captured by training datasets to build algorithms robust and accurate enough to build models suitable for clinical deployment. Existing AI models allow for automated diagnosis, prognosis, and risk stratification; however, the vast majority have been built on the adult population. For example, in the adult literature automated diagnosis, prognosis, or risk stratification using DL convolutional neural networks from parameters derived from images such as those for coronary artery disease [47], hypertrophic cardiomyopathy [46], dilated cardiomyopathy [46], restrictive cardiomyopathy [46], cardiac amyloidosis [48], myocarditis [49], Ebstein anomaly [16,17], arrhythmogenic right ventricular cardiomyopathy [16], Takotsubo [50], atrial fibrillation [51], aortic stenosis [52], and sarcoidosis [53]. Machine learning models trained on the motion of the segmented right ventricle in pulmonary hypertension, for example, have been able to predict 1 year mortality [54]. Additionally, novel DL pipelines for the screening of pulmonary arterial hypertension in adults have shown very favorable results through methods such as CMR disease features extracted by multilinear principal component analysis (MPCA) to predict the presence or absence of pulmonary arterial hypertension (PAH) [55] without the need for segmentation. Unfortunately, pediatric disease examples are deficient. Almost certainly, even with advancement in AI, there will still be a significant need for human oversight in the final interpretation of scans, particularly for complex CHD cases.

## 6. Ethical Considerations

As AI-based technology continues to grow and expand in uptake in pediatric CMR, ethical concerns will likely arise and warrant discussion and the development of guidelines to minimize patient risk. In an ideal system, AI algorithms should be designed to identify and reduce bias introduced by age, race, ethnicity, or gender, and should protect patient information and privacy by using metrics such as data anonymization and data reduction. Methods should attempt to recruit large datasets of diverse patient populations to reduce biases. In pediatric CMR, to reduce bias and misdiagnosis, large training datasets for algorithmic development are needed, highlighting the importance of cloud-based systems for multicenter collaboration to accrue large datasets. Furthermore, human overreads of models to reduce misdiagnosis should be employed as a final check. Additional key considerations when developing algorithms should include explainability, robustness, transparency, inclusion, reliability, and accuracy. Furthermore, after algorithms are built there should be ongoing efforts made to continuously test AI models for bias. All models should be publicly available for transparency and trust.

## 7. Conclusions

Overall, AI has revolutionized pediatric cardiac MRI by addressing key challenges in image acquisition, motion artifact correction, and reconstruction. These advancements improve efficiency, reduce acquisition times, and enhance diagnostic accuracy, benefiting both clinicians and patients. Compelling new research has unfolded over the past decade. As industry takes more of a foothold in translating research into clinical practice and reported use cases grow, these solutions will become more widely used across different CMR applications. While the clinical translation will most likely first be seen in adult CMR, recent pediatric publications and a few sample clinical cases seen primarily at academic conferences illustrate the potential for AI to make powerful improvements in image quality and in spatial resolution using novel DL image reconstruction techniques in pediatric CMR. Furthermore, ongoing reported innovations are not only allowing for improved image quality but also easing the training burden on technologists with standardized image protocols, supporting a better patient experience with shortened exam times, and automating many of the calculations and postprocessing tasks radiologists and cardiologists need to interpret scans. As innovation in AI for CMR applications continues, new insights into cardiac disease and treatments that were not possible before have also arisen.

## Figures and Tables

**Table 1:** Proposed potential benefits, challenges, and solutions for AI in pediatric CMR.

Metric	Potential Benefits of AI	Challenges	Ways to Overcome Challenges
Acquisition	Reduced anesthesia exposure with faster scan times. Increased patient cooperation with shorter scans. Real-time motion artifact correction.	Difficult to preserve image quality. May rely on undersampling techniques, which may be less reliable in the heterogeneous CHD population.	Combination with DL-based reconstruction techniques may preserve image quality. Multi-center collaborations. Improvements in MRI hardware.
Image Reconstruction	Shorter acquisition times with preserved image quality. Improved denoising result in higher image quality.	Limited publicly available datasets for training. Denoising and techniques remain untested in complex congenital heart disease.	Advances in synthetic dataset creation. May be able to translate techniques that are being pioneered in non-cardiac MRI. Multi-center collaborations.
Volumetrics	Faster contouring. Reduced intra- and inter-reader contouring differences.	Few algorithms trained on CHD cases. Broad anatomic variability of cases. Low numbers at single centers. Difficulties in data and intellectual property-sharing.	Government or foundation funding to create data-sharing infrastructures. Multicenter studies.
4D flow	Faster segmentation. Improved flow quantification.	Limited datasets. Long postprocessing time. Spatial resolution depends on contrast.	High-speed imaging techniques. Improved computing capacity.
